# QbD Approach-Based Preparation and Optimization of Hydrophobic Ion-Pairing Complex of Lysozyme with Sodium Dodecyl Sulphate to Enhance Stability in Lipid-Based Carriers

**DOI:** 10.3390/pharmaceutics16050589

**Published:** 2024-04-26

**Authors:** Alharith A. A. Hassan, Tamás Sovány, Krisztián Pamlényi, Martin Deák, Viktória Hornok, Edit Csapó, Géza Regdon, Ildikó Csóka, Katalin Kristó

**Affiliations:** 1 Institute of Pharmaceutical Technology and Regulatory Affairs, University of Szeged, Eötvös u. 6., H-6720 Szeged, Hungary; alharith.hassan@szte.hu (A.A.A.H.); sovany.tamas@szte.hu (T.S.); pamlenyi.krisztian@szte.hu (K.P.); deakmartin09@gmail.com (M.D.); csoka.ildiko@szte.hu (I.C.); 2 Department of Pharmaceutics, Faculty of Pharmacy, University of Khartoum, Khartoum P.O. Box 1996, Sudan; 3 Interdisciplinary Excellence Center, Department of Physical Chemistry and Materials Science, University of Szeged, Rerrich B. Sqr. 1, H-6720 Szeged, Hungary; vhornok@chem.u-szeged.hu (V.H.); juhaszne@chem.u-szeged.hu (E.C.); 4 MTA-SZTE Lendület “Momentum” Noble Metal Nanostructures Research Group, University of Szeged, Rerrich B. Sqr. 1, H-6720 Szeged, Hungary

**Keywords:** critical quality attributes, hydrophobic ion-pairing, lysozyme, quality by design, risk assessment

## Abstract

Hydrophobic ion pairing (HIP) complexation was found to be an efficient approach in modulating the release and enhancing the stability and encapsulation of hydrophilic macromolecules such as proteins in hydrophobic nano/microcarriers. The present work strives to develop and optimize the preparation of the HIP complex of the antimicrobial enzyme lysozyme (LYZ) with the ion-pairing agent (IPA) sodium dodecyl sulphate (SDS) relying on the quality-by-design (QbD) approach. The quality target product profile (QTPP) includes the achievement of maximal lipophilicity in a reversible manner to enable the maintenance of biological activity. The related critical quality attributes (CQAs) were defined as complexation efficacy, complex stability, enzyme recovery and activity. Three risk assessment (RA) tools were used to identify and rank the critical process parameters (CPPs) and critical material attributes (CMAs). From this assessment, the pH of the medium, LYZ:SDS molar ratio and drying conditions were determined as high-risk factors that need to be investigated. To the best of our knowledge, for the first time, electrostatic titration was used as a smart approach to determine the optimum molar ratio at different pH values. Based on the predefined CQAs, pH 8 with an LYZ/SDS molar ratio of 1:8 was found to be the optimal condition for complexation efficiency and recovery (%) of a biologically active enzyme. A cost-effective drying process based on a ventilated oven was developed, which resulted in complex qualities comparable to those obtained by the commonly used freeze-drying method. In a nutshell, the optimum conditions for the preparation of the LYZ/SDS HIP complex were efficiently facilitated by the rational application of QbD principles and the utilization of efficient electrostatic titration and ventilated oven-drying methods.

## 1. Introduction

The share of biopharmaceutical therapies such as peptides and proteins in the pharmaceutical market is growing rapidly by virtue of their potency, high selectivity and low toxicity. However, chemical degradation, mucosal binding, harsh conditions in the gastrointestinal (GI) tract and/or rapid blood clearance represent barriers that could prevent proteins and peptides from reaching their targets [1,2,3,4,5,6].

Different approaches have been used to deliver these peptides and proteins nonparenterally, in particular via the oral route, by encapsulating them into mostly colloidal delivery systems to improve performance, overcome biological barriers and reduce dose frequency. However, several shortcomings have arisen from these strategies including low drug loadings, considerable losses, denaturation and aggregation of fragile active pharmaceutical ingredients (API), limitations associated with batch-to-batch reproducibility and manufacturing scale-up [2,3,4,5,6,7,8,9,10,11].

One of the strategies that have been employed to improve the encapsulation in and/or modulate the release profile of hydrophilic molecules, some hydrophilic antibiotics and peptide/protein APIs from micro/nanocarriers is hydrophobic ion pairing (HIP) complexation. In this approach, the ionizable functional groups on a hydrophilic molecule are engaged stoichiometrically in ionic interactions with an oppositely charged counterion containing one or more hydrophobic moieties at a suitable pH. In this complexation, e.g., in the case of peptide/protein, the charge of amino acids is neutralized in a reversible manner resulting in a dramatic reduction in aqueous solubility of the molecule of interest and consequently increasing the hydrophobicity and enhancing the ability to be encapsulated in a colloidal carrier using techniques developed for hydrophobic molecules, for instance, the double emulsification method. Other possible advantages of this complexation are the enhanced capability of protein transportation across the mucosal membrane and the improved conformation stability of the protein in the presence of organic solvents. On top of that, this technique is simple, inexpensive and not involving chemical modifications that could impart efficacy and safety issues [1,2,3,5,6,7,9,10,11,12,13,14,15].

Lysozyme (LYZ) is a naturally occurring single-chain polypeptide known for its antimicrobial activity by enzymatically cleaving a glycosidic linkage of peptidoglycan present in bacterial cell walls leading to cell death. LYZ can be found in many biological organisms, and the chicken egg white represents a rich source for it. It is considered a part of innate immunity and an important defense mechanism. In addition to bacteria, the antimicrobial activity of LYZ has been reported to be effective against fungi, protozoa and viruses. It consists of 129 amino acids cross-linked with four disulfide bridges with a molecular weight of 14.4 kDa. It is a basic protein positively charged below its isoelectric point (pI ≈ 11.3). The enzyme is active over a broad pH range [4,16,17,18,19,20,21]. LYZ is commonly employed as a model protein in several pharmaceutical formulation studies including formulation of macromolecular hydrophilic drugs in nonparenteral dosage forms [3,5,6,8,11,22,23].

Different research groups have prepared and tried to optimize HIP complexes of LYZ with amphipathic molecules such as sodium dodecyl sulphate (SDS) and sodium oleate. These studies investigated a number of factors, such as the pH of the medium and peptide/counterion ratio, that could affect the efficiency of this complexation and the biological activity of the peptide [3,5,7,11]. In one study, to prepare the HIP complex of LYZ with SDS, different pH values from 3 to 11 using water as a medium were investigated. The author found that the optimum pH was 8 and determined the optimum molar ratio of LYZ to SDS to be 1:7.4 [24]. In another work, Yoo et al. selected 1:6 LYZ/SDS as the optimum molar ratio and 10 as the optimum pH using the Tris buffer as a medium for the preparation [11]. In recent studies, lower pH values in the acidic range, namely pH 4.5 and 2, were reported as optimum values to achieve high complexation efficiency and higher optimum molar ratios of 1:12 and 1:14, respectively [5,7].

These reports showed discrepancies and variations in the determined optimum medium pH and molar ratio as well as achieved complexation efficiency and recovered enzyme activity. Moreover, different media, pH ranges and values and experimental specifications were used that could lead to these variabilities. It appears that there are gaps that need to be filled to understand this complexation process and to maximize output.

For the above-mentioned reasons, the main aim of this work was to establish a comprehensive investigation of different factors affecting the preparation of LYZ and the SDS HIP complex. To achieve this goal, quality-by-design (QbD) principles were employed. The QbD is a knowledge-based paradigm aiming to manage the quality of the product according to predefined expectations by employing a variety of tools such as risk assessment (RA) [25,26,27]. RA was used in this study to identify different variables and prioritize them according to their expected effects. This assessment included defining the quality target product profiles (QTPPs) of the product which are the quality, efficacy and safety properties of a product. Based on the QTPPs and previous knowledge, the critical quality attributes (CQAs) were derived which include physical, chemical and/or biological properties. Critical material attributes (CMAs) and process parameters (CPPs) that could influence the CQAs were also determined and ranked according to their degree of impact based on the literature, previous experience and preliminary study [25,27,28]. 

Based on the output of this assessment, the investigations were devoted to the identification of the optimal combination of high-risk factors using a cost-effective methodology that would provide a high complexation yield in a reversible manner, which would provide increased stability in the lipophilic environment but preserved enzymatic activity of the API in a biological environment. The new drying method may also improve the cost-effectiveness of the complexation process.

## 2. Materials and Methods

### 2.1. Chemicals

Lyophilized powder of LYZ from chicken egg white (Mw = 14.4 kDa) and SDS were purchased from MedChemExpress (Monmouth, NJ, USA) and EGIS (Budapest, Hungary), respectively. Lyophilized cells of *Micrococcus lysodeikticus* were purchased from Sigma-Aldrich (St. Louis, MO, USA) and sodium chloride Ph.Eur. was brought from VWR International (Leuven, Belgium). Chemicals were used for pH adjustment. For the preparation of different solutions, double-distilled water was used.

### 2.2. Risk Assessment

As mentioned above, RA is one of the most important QbD tools to identify different factors affecting the output of a process. This assessment was carried out based on a literature survey, preliminary experiments and the experience of our research group. The assessment was commenced by defining the QTPPs and selecting the CQAs of the product. Then, CMAs and CPPs that could have an impact on the CQAs were identified and ranked in terms of severity scores.

To perform the RA, three quality tools were employed; namely a cause-and-effect (Ishikawa) diagram, a risk estimation matrix (REM) and a Pareto chart. A cause-and-effect diagram was constructed to identify and organize possible causes that affect the CQAs of the HIP complex and reveal the relationships between them.

Both the Pareto chart and REM helped in building up and showing the relationships between CMAs and CPPs, and CQAs and the factors with the higher influence were determined. Consequently, the experimental investigations were focused on those high-risk factors. The LeanQbD software (QbD Works LLC, Fremont, CA, USA/Version 1.3.6., 2014) was employed to aid in performing both analyses by evaluating the risk severity scores to rank and prioritize different factors affecting the preparation of the HIP complex.

### 2.3. Determination of the Optimum Molar Ratio

Particle charge detector (PCD) (PCD02 model, Mütek GmbH, Herrsching, Germany) was employed to determine the optimum molar ratio of LYZ to SDS at four pH values of 4, 6, 8 and 10. During measurements, the 7 µM SDS solution was added in a dropwise manner to 10 mL of a 2 mg/mL LYZ solution contained in the cell of the PCD. Charge compensation points were determined in all cases (when the streaming potential values reached zero). The optimum molar ratio for complexation was calculated based on the volume of SDS consumed in the titration to reach the charge compensation point applying the results of at least three parallel measurements. 

### 2.4. Preparation of the HIP Complex

The procedure to prepare the HIP complex was adopted from a number of previous reports with some modifications based on our preliminary study [5,7,11]. LYZ (in a concentration of 4 mg/mL) and SDS (in concentration according to the finding of optimal molar ratio determination) were separately dissolved in phosphate buffer (pH 6.8) and the pH of the solutions was adjusted to the required value (e.g., 4, 6, 8 or 10) using 1 M HCl or 1 M KOH to ensure that the complexation takes place at the required pH value. Then, equal volumes of both solutions were added into Eppendorf tubes, mixed by pipetting, and allowed to stand for 30 min. Consequently, the final peptide concentration of 2 mg/mL was used in all experiments. After that, the samples were centrifuged at 15,000 rpm for 15 min at room temperature. The supernatant was removed, and the precipitated complex was dried.

For the samples dried by freeze drying, Scanvac Coolsafe laboratory freeze dryer (LaboGene, Lillerørd, Denmark) was used at −20 °C for 24 h under a pressure of 1.3 Pa and then kept at 25 °C for 24 h for secondary drying to obtain lyophilized powder [6].

### 2.5. Determination of the Dissociable Amount of LYZ from the HIP Complex by NaCl

A volume of 2 mL of 0.1, 0.5, 1.0 and 1.5 M NaCl solutions in phosphate buffer were added to dried HIP complexes and incubated in Eppendorf tubes at 8 °C for 24, 48, 72 and 168 h. After that, the solutions were centrifuged at 15,000 rpm for 15 min at room temperature. Then, the amounts of the dissociated LYZ (recovery%) and their enzymatic activities were measured. The effects of three input factors, namely the pH at which the complex was prepared, the concentration of the NaCl solution and the incubation period on the percentage of the recovered LYZ and its enzymatic activity were investigated and evaluated statistically using the Statistica software, version 13 (TIBCO Software Inc., PaloAlto, CA, USA). Differences at *p* < 0.05 were considered significant using a factorial analysis of variance (ANOVA) F-test.

Similarly, the cumulative recovered amount of LYZ from the complex was calculated. However, after centrifugation and withdrawing the supernatant solution, a fresh NaCl solution was added again to the same Eppendorf tubes containing the complex to be incubated at 8 °C till the next point of measurements. After several repetitions of this process, the sum of the recovered LYZ was calculated.

### 2.6. Determination of the Complexation Efficiency and Recovered LYZ

The amount of the complexed LYZ was indirectly determined by measuring the concentration of the free LYZ in the supernatant using a UV spectrophotometric method at λ_max_ = 281 nm (determined experimentally) [24]. The calculations were based on equation *y* = 2.452*x*, where *y* is the absorbance and *x* is the concentration of the solution in mg/mL. The amount of the complexed LYZ was calculated by the following equation:

(1) $$Complexation\;efficiency\left(\%\right)=\frac{Initial\;amount\;of\;LYZ-Amount\;of\;LYZ\;in\;the\;supernatant}{Initial\;amount\;of\;LYZ}\times 100\%$$

The amount of the dissociated LYZ was determined by the same UV spectrophotometric method at λ_max_ = 281 nm using the following equation:

(2) $$Recovery\left(\%\right)=\frac{Amount\;of\;LYZ\;in\;the\;supernatant}{Initial\;Amount\;of\;LYZ\;in\;the\;complex}\times 100\%$$

### 2.7. Biological Activity of the Dissociated Lysozyme

The biological activity of the recovered LYZ was measured using an enzymatic assay test [29]. A lyophilized *Micrococcus Lysodeikticus* bacterial suspension in a phosphate buffer adjusted to pH 6.24 at 25 °C was used as a substrate. The enzymatic assay was based on the spectrophotometric rate determination of absorbance at λ = 450 nm (A_450_, light path = 1 cm) vs. time which is based on the following reaction:

*Micrococcus lysodeikticus* Cells (Intact) → Lysozyme → *Micrococcus lysodeikticus* Cells (Lysed)

The enzymatic activity of the LYZ in different samples was expressed in a percentage (Enzymatic activity %) compared to an LYZ reference solution.

### 2.8. Development and Optimization of the Drying Method

A method of drying for the LYZ/SDS HIP complex was developed using a ventilated oven (Memmert GmbH + Co. KG, Buechenbach, Germany). The effects of two input factors, namely the temperature and the fan (airflow) speed, were investigated using a 2^2^ full factorial design (Table 1). The slope of the exponential decay curve (k) which is directly proportional to LYZ enzymatic activity was selected as a response in this evaluation. Six replicates for each run were performed, and the building of the design, analysis of the data, and drawing of the graphics were executed using the statistical program Statistica, version 13 (TIBCO Software Inc., Palo Alto, CA, USA).

### 2.9. Fourier Transform Infrared Spectroscopy (FTIR) Analysis

To characterize the complex formation and secondary structure of LYZ in the HIP complexes prepared at the optimum pH and molar ratio and dried by the oven and freeze-drying methods, the infrared spectra of the LYZ, SDS, oven-dried HIP and freeze-dried HIP complexes were obtained using an FT-IR spectrophotometer (Avatar 330 FT-IR, Thermo Fisher Scientific Inc., Waltham, MA, USA) with the potassium bromide pressed disc method. The samples were scanned for absorbance in the wavelength range of 600–4000 cm^−^^1^. The spectra were obtained from 128 scans with a spectral resolution of 4 cm^−^^1^ and applying H_2_O and CO_2_ corrections. SpectraGryph software (version 1.2.15.; Dr. Friedrich Menges Software, Entwicklung, Germany) was used to evaluate the results.

### 2.10. Differential Scanning Calorimetry (DSC)/Thermogravimetric Analysis (TGA) Measurements

The thermoanalytical behavior of the HIP complexes prepared at the optimum pH and molar ratio and dried by the oven and freeze-drying methods was investigated using a Mettler-Toledo TGA/DSC1 instrument (Mettler Toledo GmbH, Greifensee, Switzerland). The thermograms of LYZ, SDS, oven-dried HIP complex and freeze-dried HIP complex were obtained by placing 7–11 mg of samples in sealed aluminium pans with 2 holes on top (100 µL) and inserting them into the calorimeter. The starting temperature in the measurement was 25 °C and the end temperature was 500 °C. The heating rate was 10 °C/min. The samples were investigated under a flow of anhydrous nitrogen with a flow rate of 50 mL/min. STAR^e^ SW 16.30 software was used to evaluate the results and each measured sample was measured minimum twice.

## 3. Results and Discussion

### 3.1. Preliminary Study

#### 3.1.1. Determination of Suitable Solvent and LYZ Solution Concentration

In the previous studies, different solvents, for example, water and phosphate buffer, were used to prepare LYZ solutions at different concentrations [5,7,24]. Consequently, different LYZ solutions were prepared in distilled water as a solvent at different concentrations, namely 10, 5 and 2 mg/mL. The initial pH values of these solutions were 3.48, 5.07 and 5.95, respectively. When the pH values of these solutions were adjusted to different pH values from 2 to 11, LYZ started to precipitate at pH values higher than the initial pH values and at a faster rate at higher LYZ concentrations. For instance, in the 10 mg/mL LYZ solution, LYZ precipitated in all solutions from pH 5 to 11 and the amount increased with increasing pH. At this concentration, the precipitation happened in less than 10 min, while in the 2 mg/mL solution, it took around 24 h to see the precipitation.

The same experiment was repeated using phosphate buffer (pH 6.8) as a solvent using the same concentrations as before. At 10 and 5 mg/mL, the precipitation happened again within 1 h of observation but at higher pH values, and it was less dense and slower compared to that in water. At 2 mg/mL concentration, the precipitation did not happen at any of the pH values within 1 h of observation. After 24 h, there was very limited precipitate at all pH values, and they were similar in the amount except at pH 11 in which the solution was clear even after 24 h.

Based on these observations, phosphate buffer was selected as a solvent in the following experiments and 2 mg/mL was the final concentration of LYZ in the complexation vessel.

#### 3.1.2. HIP Complexation

The HIP complex of LYZ with SDS as an ion pairing agent (IPA) was prepared at different pH values from 2 to 11 using the reported molar ratio of one to six, respectively [11]. Variable complexation efficiencies were obtained ranging from a minimum of pH 3 to a maximum of pH 8 (see Figure S1 in Supplementary Material). Because LYZ gradually becomes more positively charged moving towards lower pH values apart from its isoelectric point, it was initially expected that more complexation would be obtained at reduced pH values [11]. This observation could be justified by the fact that due to a higher degree of ionization of LYZ at reduced pH values, higher ratios of SDS are required to cause and achieve a high degree of complexation and precipitation. At pH 11, the complex was not obtained as it was almost equal to the isoelectric point of LYZ (pI ≈ 11.3). It can be concluded from this experiment, in addition to what was obtained from the literature, that the pH of the solution and the LYZ: IPA molar ratio are critically affecting the complexation and the yield of this process. Therefore, these factors were included in the risk assessment.

### 3.2. Risk Assessment

#### 3.2.1. Cause-and-Effect Diagram

**Figure 1:**
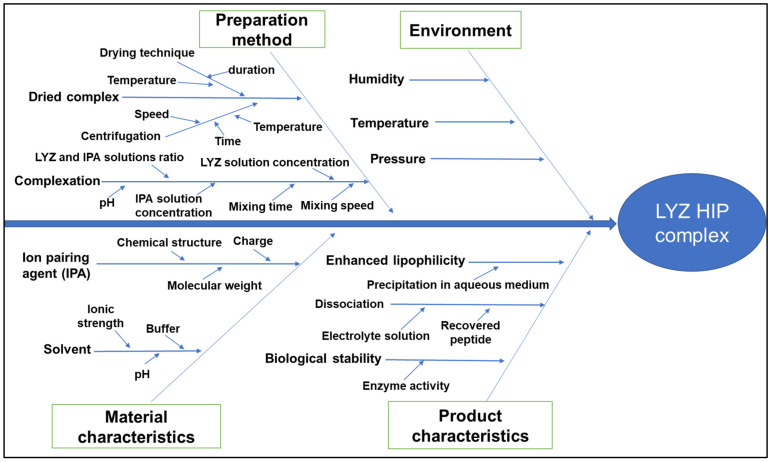
Cause-and-effect diagram showing the identified key parameters and the effect–cause relationships among CMAs, CPPs and CQAs for LYZ HIP complex preparation.

As mentioned before, RA is one of the most important tools of the QbD approach. This assessment was commenced by a comprehensive revision of the reports dealing with the HIP complexation in general and of LYZ in particular. In addition to that, the pilot experiment reported in the previous section and the experience of our research group helped in identifying the possible parameters that could affect the quality of the prepared complex. A cause-and-effect diagram was built, showing the root causes that affect the quality of the LYZ HIP complex (Figure 1).

#### 3.2.2. Definition of the QTPPs and Determination of the CQAs and REM

As an essential part of the RA process, the QTTPs and CQAs of peptide/protein HIP complex were defined both from previous reports and the preliminary study, and the data are listed in Table 2. In addition, the interrelationships between QTTPS and CQAs were established and rated by assigning low (L), medium (M), and high (H) values for each parameter (Figure S2). A 1(L)-2(M)-3(H) scale was utilized for probability rating. After defining the CQAs of the protein HIP complex, the CMAs and the CPPs of the preparation method were determined. This was followed by building up the REM showing the impact of CMAs and CPPs on the CQAs of the complex (Figure S3).

#### 3.2.3. Pareto Analysis

**Figure 2:**
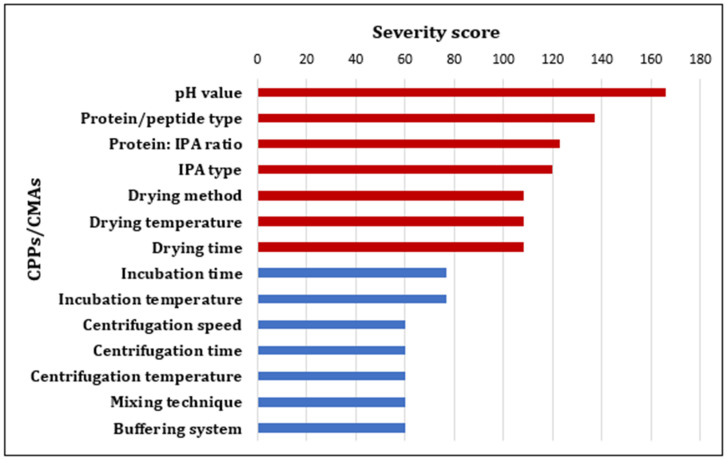
Pareto chart showing the ranking of the critical material attributes (CMAs) and critical process parameters (CPPs) for the preparation of the peptide/protein HIP complex (red color: high risk factors, blue color: low risk factors).

A Pareto chart ranking the CQAs of the HIP complex was generated (Figure S4). Based on the REM, another Pareto chart was built ranking the impact of different CMAs and CPPs on the CQAs in terms of severity scores (Figure 2). Based on the REM and Pareto analysis, pH value, type of protein, type of IPA, protein/IPA molar ratio and the drying parameters (drying method, temperature and time) were revealed as high-risk factors affecting the HIP complexation process. In this study, the protein of interest was LYZ, and SDS was selected as an IPA to form such a complex. SDS, which is a well-known anionic surface-active agent, was commonly used in several studies to make complexes with peptides/proteins and with LYZ in particular. It has been reported that SDS forms stable complexes with proteins that increase their stability and protect their secondary and tertiary structures against thermal denaturation [30,31,32]. Owing to a hydrophilic head sulphate group (SO_4_^2−^) which is classified as a kosmotropic ion (water structure maker) on the Hofmeister ion series, SDS tends to stabilize the native fold structure of the protein, resulting in salting-out behavior [33]. Moreover, SDS has a hydrophobic tail that helps in achieving high complexation efficiencies with proteins [1]. Therefore, the investigations were devoted and focused on the remaining high-risk factors, namely the peptide/IPA ratio, the pH of the medium and the drying method factors. The responses of these evaluations were the predetermined CQAs in terms of complexation efficiency (%), enzymatic activity (%) and recovery (%). Lower-risk factors were kept constant in all experiments, and their levels are shown in the methodology section.

### 3.3. Optimization of the LYZ/SDS Molar Ratio in the HIP Complex

In this study, the optimum molar ratio of the protein and IPA was determined experimentally at four different pH environments. In polypeptide molecules, the degree of ionization of amino acid residues is essentially determined by the pH value [5]. As noticed from the preliminary study, using a fixed molar ratio of 1:6 of LYZ to IPA resulted in lower complexation efficiencies at lower pH values compared to the higher values. This can be attributed to a higher degree of ionization in an acidic environment and, consequently, higher ratios of SDS are required to achieve a high degree of complexation. Therefore, an optimum LYZ/SDS ratio needs to be determined at each selected pH value to maximize the complexation, and that requires tedious work and numerous experiments.

**Figure 3:**
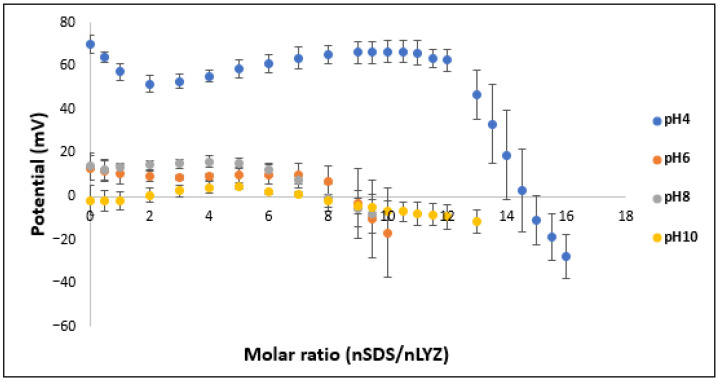
Titration curve representing the change in streaming potential (mV) upon the addition of small increments of a 7 µM SDS solution to a 2 mg/mL LYZ solution at pH 4, 6, 8 and 10. The intersection of the curves with a zero potential line represents the average SDS/LYZ molar ratios of three measurements at the neutralization point.

To the best of our knowledge, this is the first time that electrostatic titration was used to determine such a ratio by employing the PCD technique. This smart approach helped in saving time and resources to determine the optimum molar ratios of both complex components in order to maximize the yield of the complexation process. As the HIP complexation is based mainly on electrostatic interactions, this electrostatic titration enabled the determination of the point of complete neutralization of the surface charge of the LYZ molecule by the IPA at the corresponding pH value (Figure 3). It is apparent that as we moved from the isoelectric point of LYZ, a greater amount of the IPA was required; for instance, the ratio of SDS at pH 4 was almost double that at pH 10, reflecting that more ionizable groups are available for this interaction which is in agreement with a previous report [5]. 

**Figure 4:**
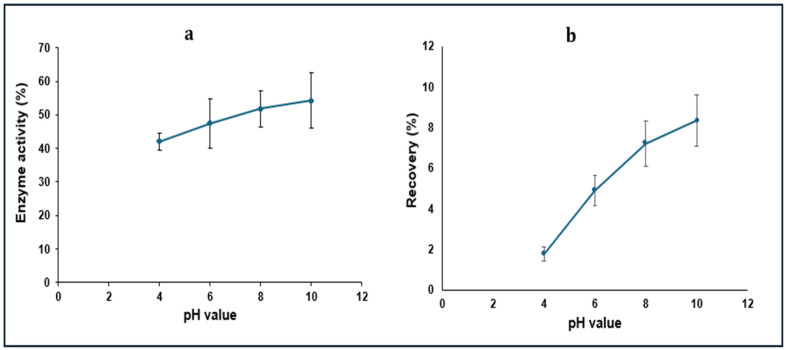
Enzymatic activity (%) (**a**) and the percentage of the dissociated LYZ (**b**) from the complexes prepared at pH 4, 6, 8 and 10 upon addition of the 0.5 M NaCl solution and incubation for 24 h (average of six measurements).

To confirm the results of the titration experiment, the obtained molar ratios, namely 14.6, 9.2, 8 and 7.5 SDS/LYZ at pH 4, 6, 8 and 10, respectively, were used experimentally to prepare the complex. A high yield of complexation above 95% was obtained for complexes prepared at pH 4, 6 and 8 (Figure S5). Lower complexation efficiency was obtained at pH 10, which can be justified by the closeness of this value to the isoelectric point of this peptide; consequently, a lower number of ionizable groups is available for the complexation interaction. Moreover, the enzymatic activity of the complexed LYZ was measured after inducing dissociation upon the addition of 2 mL of a 0.5 M NaCl solution to the dried complexes and incubation for 24 h (Figure 4a). In contrast to the complexation efficiency results, the complex prepared at pH 10 showed the highest enzymatic activity while that prepared at pH 4 showed the lowest value. This can be justified by the recovery % outputs (Figure 4b) as they showed the same pattern; therefore, the lower enzymatic activity obtained from LYZ prepared at pH 4 was due to the lower dissociated amount of this enzyme. However, as the complexation yield at pH 10 was much lower than others, this pH value was excluded from further investigations.

### 3.4. Optimization of HIP Complex Dissociation Conditions

**Figure 5:**
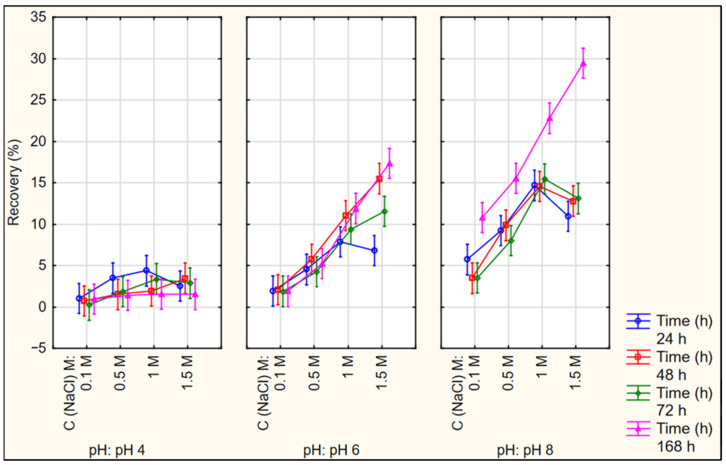
Recovered LYZ (%) from the HIP complex prepared at pH 4, 6 and 8 after incubation in different NaCl molar concentration solutions (average of three replicates).

One of the most important criteria of the HIP complexation is the reversibility and liability to dissociate into its individual components in the presence of an excess amount of oppositely charged ions. To achieve and maximize this dissociation, different concentrations of NaCl solution were added to complexes prepared at pH 4, 6 and 8. The effects of the complex preparation pH, the concentration of the NaCl solution and the incubation time on the dissociation process were investigated. Figure 5 and Figure S6 show the obtained recovery (%) and enzymatic activity (%) of the three complexes. 

In general, the results obtained for both responses from the complex prepared at pH 4 were much lower than those prepared at the other two pH values. Also, it could be observed from the graphs that the highest enzymatic activity and recovery (%) were at the 1 M NaCl and 24 h incubation period in case of pH 4, while the highest response values were at the 1.5 M solution after 168 h of incubation in both complexes, prepared at pH 6 and 8. All effects (main effects and interactions) were statistically significant (*p* < 0.05). As a general trend, increasing the pH, NaCl concentration and incubation time resulted in increased enzymatic activity and recovery (%). Higher enzyme activity can be correlated partially with the higher obtained dissociated amount of the protein. The differences in recovery (%) from complexes prepared at the same pH value upon changing the incubation time were not significant except for pH 8 and 168 h of incubation. In complexes prepared at the same pH value, increasing NaCl concentration resulted generally in increased recovery (%). The differences were significant (*p* < 0.05) at pH 6 and 8 but were not significant (*p* > 0.05) for the complexes prepared at pH 4. As the values of both responses obtained from complexes prepared at pH 4 were much lower than those prepared at pH 6 and 8, the former pH value was excluded from further investigations.

### 3.5. Cumulative Recovered Amount of LYZ Dissociating from the HIP Complex

**Figure 6:**
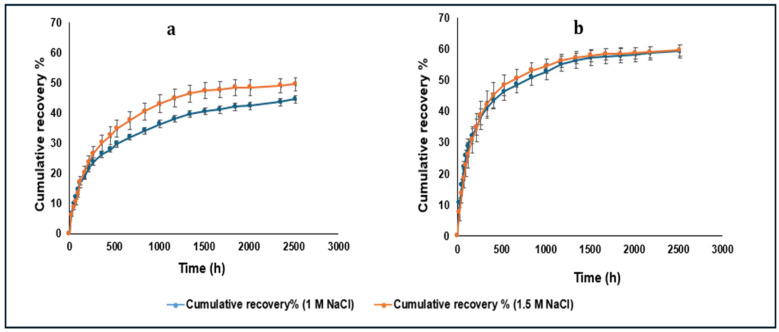
Cumulative recovered amount of LYZ from the complexes prepared at pH 6 (**a**) and 8 (**b**) upon addition and incubation in 1 M and 1.5 M NaCl solutions (average of five measurements).

The obtained cumulative amount of LYZ resulted from the dissociation of the HIP complex prepared at pH 6 and 8 was determined. Based on the results of the previous section, 1 M and 1.5 NaCl solutions were used to induce the dissociation in both cases. The cumulative recovered amount of LYZ from the complex prepared at pH 8 after approximately 100 days was higher than that obtained from the pH 6 complex in both concentrations of NaCl solutions (Figure 6). Factorial ANOVA analysis showed a significant (*p* < 0.05) difference in the recovered LYZ amount between pH 6 and 8 prepared complexes. In the case of the pH 6 prepared complex (Figure 6a), the 1.5 M NaCl solution resulted in significantly (*p* < 0.05) higher recovery (%) compared to the 1 M solution, while in the case of pH 8 (Figure 6b), both solutions caused an almost similar dissociation degree. Interestingly, the dissociated protein was enzymatically active even after 100 days of incubation in both cases (Figure S7). The maximum enzymatic activity (%) of LYZ recovered from the complex prepared at pH 8 (≈26%) was higher than that recovered from the pH 6 complex (≈20%). Based on the results of this experiment and the previous one, pH 8 was the pH of choice in terms of complexation efficiency (%) as well as recovery (%). As there was no significant difference in the recovered amount of LYZ from the complexes prepared at pH 8 using 1 M and 1.5 M NaCl solutions, the 1 M solution was selected as the optimum NaCl solution concentration to maximize the dissociation of the LYZ/SDS HIP complex and to be used in the following experiments.

### 3.6. Effect of the Drying Process on the Characteristics of the LYZ/SDS HIP Complex

The effect of the drying technique on the quality of the prepared HIP complex of LYZ with SDS was studied using two drying techniques, namely ventilated oven and the freeze-drying technique. In all previous reports, freeze-drying was used to obtain such a dried complex [7,11]. To the best of our knowledge, this is the first time an optimized ventilated oven method was proposed to obtain a dried LYZSDS HIP complex. This proposal was driven by the superiority of the latter technique in terms of energy consumption and time as 2–3 h were needed to obtain a dried complex compared to about 48 h in the case of freeze drying. Moreover, LYZ has relatively higher thermal stability compared to other proteins and peptides, and it was reported that binding with detergents increases further stability against thermal denaturation [24,31].

Two levels of temperature and airflow speed were investigated in a full factorial design to determine the optimum conditions for drying of the HIP complex by the ventilated oven (Table 1). The following polynomial equation of the two-way interaction model describes the effects of the drying factors on the enzymatic activity of LYZ:

K = 0.0057 − 0.0002*x*_1_ − 0.0006*x*_2_ − 0.0001*x*_1_*x*_2_ (3)

The statistical parameters of this model are R^2^ = 0.59188, Adj. R^2^ = 0.53066, and MS residual = 0.0000003.

**Figure 7:**
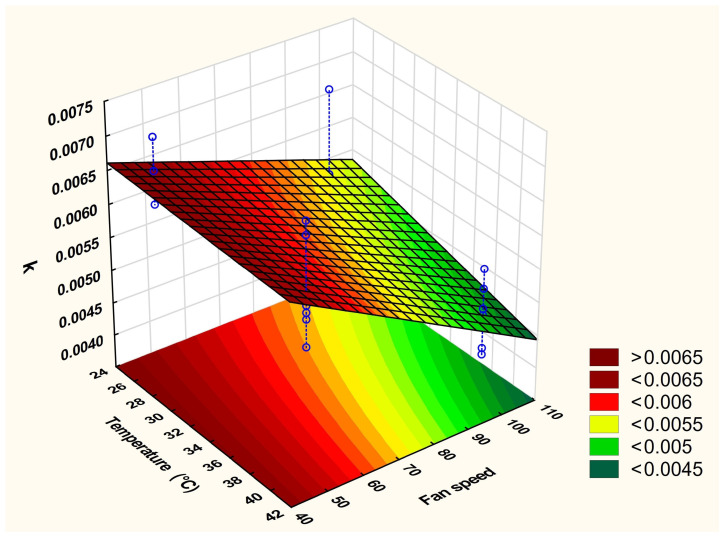
Fitted surface plot of the two-way interaction model of the two factors of two-level full factorial design investigating the effects of the applied temperature and fan speed on the enzymatic activity of LYZ dissociated from the oven-dried complex.

The speed of the airflow (*x*_2_) significantly (*p* < 0.05) affected the enzymatic activity of LYZ while the effect of the drying temperature (*x*_1_) within the used range was not significant. This can be seen from the surface plot (Figure 7), as increasing the speed of the fan led to an apparent reduction in the enzymatic activity while the drop in the enzymatic activity upon increasing the temperature from the lower to the higher level at a fixed fan speed was limited. Based on this output and because both factors are inversely proportional to the enzymatic activity, a fan speed of 50 and a 25 °C temperature were selected as the optimum drying conditions in the ventilated oven to dry the LYZ/SDS HIP complex.

**Figure 8:**
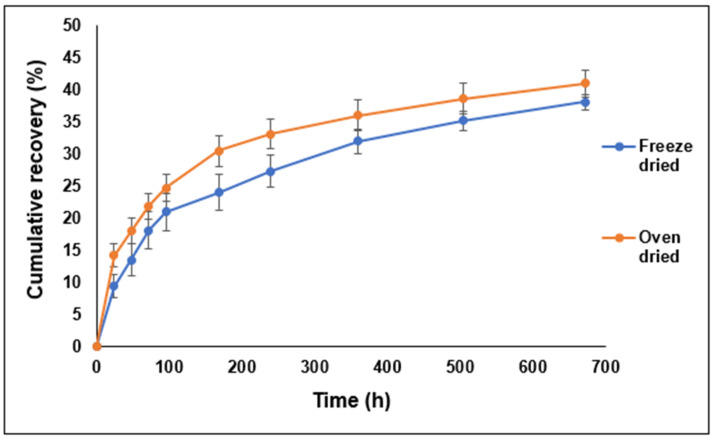
Cumulative recovery (%) of LYZ dissociated from the complexes prepared at pH 8 and dried by the freeze-drying and ventilated oven technique upon addition and incubation in 1 M NaCl solution (average of six measurements).

**Figure 9:**
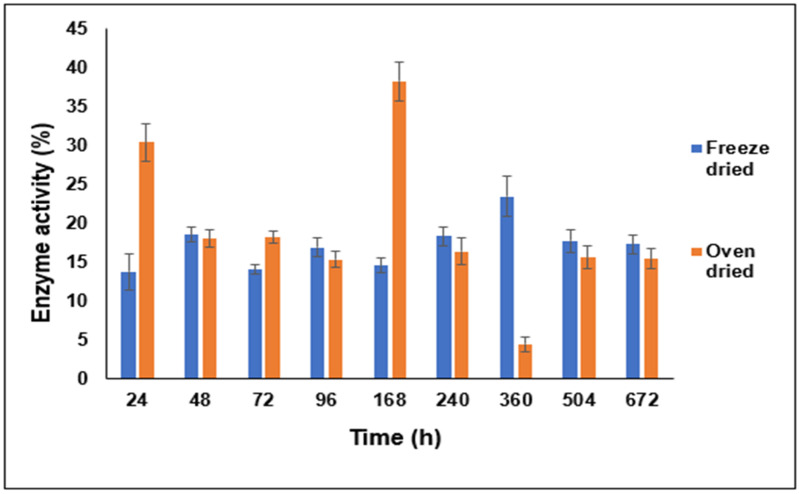
Enzymatic activity (%) of LYZ recovered from the HIP complexes prepared at pH 8 and dried by freeze drying and the ventilated oven technique and collected at different time points upon addition and incubation in 1 M NaCl solution (average of six measurements).

In opposition to our expectations, the oven-dried complex produced higher cumulative recovery (%) compared to the freeze-dried one, though the dissociation patterns look similar (Figure 8). Figure 9 shows that the protein dissociated from the complex was enzymatically active in both cases even for samples collected after four weeks of incubation. These variations in the enzymatic activity among different time points can be partly attributed to the variability in the amounts of the dissociated enzyme. Interestingly, the oven-dried complex showed a higher maximum recorded enzyme activity (%) value of the dissociated LYZ compared to the freeze-dried one. 

#### 3.6.1. Fourier Transform Infrared Spectroscopy (FTIR) Analysis 

**Figure 10:**
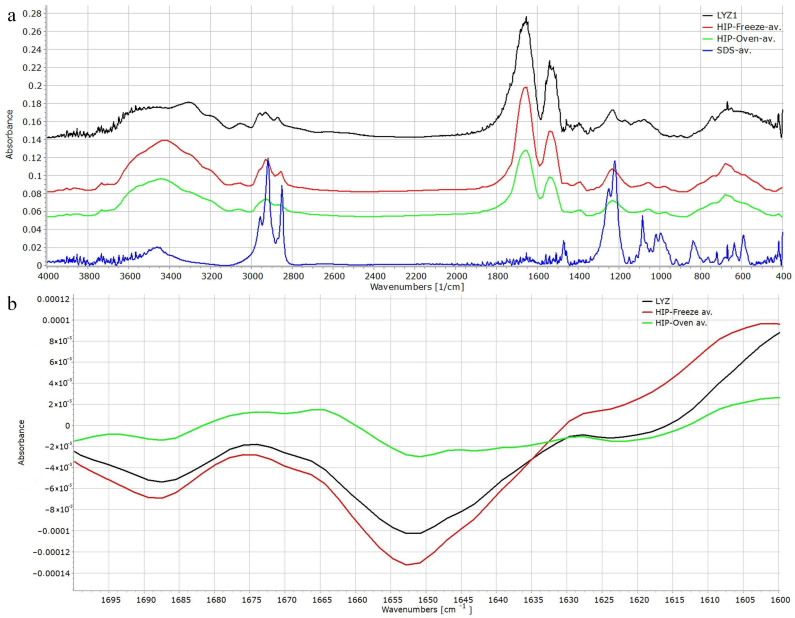
FTIR spectra of SDS, LYZ, freeze-dried HIP and oven-dried HIP complex of LYZ and SDS. (**a**) The second derivative of the amide I peak of LYZ, freeze-dried HIP and oven-dried HIP complex of LYZ and SDS (**b**).

The characteristic peaks of LYS, the C-O stretching at 1231 cm^−1^, the Amide II band at 1539 cm^−1^, the Amide I band at 1652 cm^−1^, the stretching vibrations of the CH backbone at 2876, 2934 and 2961 cm^−1^, the NH stretching at 3053 cm^−1^ and the OH stretching at 3303 cm^−1^, and similarly the CH stretching of the dodecyl backbone of SDS 2853, 2919 and 2957 cm^−1^ may be found mostly in unchanged positions in the complexed samples (Figure 10a). However, the slight right shift of the asymmetric and symmetric S=O stretching from positions 1019 and 995 cm^−1^ to 1008 and 930 cm^−1^, respectively, may be indicative of the weak interactions formed during the complexation reactions. 

If the second derivative of the amide I band of the native and HIP complexed enzymes is checked, it can be seen that the deep characteristic valley at 1651 cm^−1^ and the smaller valleys at 1622 and 1687 cm^−1^ and furthermore at 1670 cm^−1^ belonging to α-helix, β-sheet and β-turn structures, respectively [34], can be found in unchanged positions, which also confirms that there are no considerable structural changes as a result of the complexation or the drying process. 

#### 3.6.2. Differential Scanning Calorimetry/Thermogravimetric Analysis

**Figure 11:**
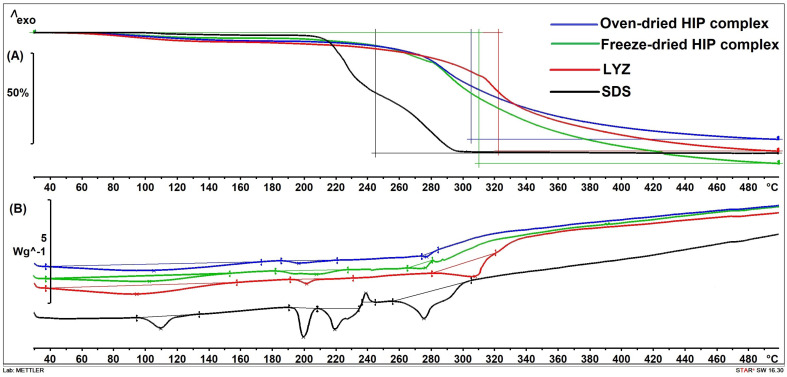
Thermograms of SDS, LYZ, freeze-dried HIP and oven-dried HIP complex of LYZ and SDS: (**A**) TGA thermogram, (**B**) DSC thermogram.

Based on visual appearance, the oven-dried complex was in the form of a colorless transparent film, while the freeze-dried one was white. This could be due to the different arrangement of the molecules, and it is known that the freeze-drying process usually results in porous amorphous powders [23]. Also, this could result in different thermoanalytical behavior of the dried materials and impart variations in solid-state stability, especially in the case of fragile compounds like proteins. Consequently, DSC, which is a well-established analytical procedure, was used to monitor solid-state transformations of complexes prepared by both drying methods along with TGA data to obtain information about solid-state stability and decomposition [17]. Figure 11 shows the thermograms of LYZ, SDS, oven-dried and freeze-dried complexes, and the peaks and the mass losses upon decomposition are listed in Table 3. Three main events appear on the SDS thermogram starting with an endothermic peak at around 110 °C reflecting the removal of water which appears as a mass loss on the TGA curve. The peak at 199.48 °C is the melting point of SDS, which is a little bit higher than what was mentioned by Gedawy et al. followed by decomposition peaks [35]. Pure LYZ showed a broad endothermic peak up to around 145 °C corresponding to the mass loss on the TGA curve reflecting the loss of strongly bonded shell of water molecules important to stabilize the native conformation of the protein. The denaturation peak appeared at 201.59 °C, which is in line with what was reported before (≈200–202 °C) [17,23]. 

A thorough revision of the literature revealed that this complexation has been studied before using DSC and/or TGA. A similar broad peak observed with the LYZ thermogram was seen in the case of both complexes reflecting dehydration as it could be understood from the TGA measurements. However, in the case of the oven-dried complex, this evaporation started later and ended at a temperature higher than that observed with native LYZ and the freeze-dried complex. This slower evaporation could be argued as a stronger binding of water molecules to the protein and, consequently, higher stabilization of the native conformation of LYZ. A freeze-dried complex thermogram shows a broad endothermic peak with the onset at around 199 °C and the endset at around 224 °C. In fact, this broad peak could be seen as two peaks overlapping with each other as the melting of SDS and denaturation of LYZ occur at temperatures close to each other. However, a smaller endothermic peak at a lower temperature was seen on the oven-dried complex thermogram, which could be due to the slight shifting of the denaturation peak. Peaks like the last decomposition endothermic peak on the SDS thermogram at around 275 °C could be seen on both complexes’ thermograms but were smaller.

## 4. Conclusions

In this study, the HIP complex of LYZ with SDS was developed and optimized by employing QbD principles. With the help of three RA tools, risk factors that affect the predefined CQAs (e.g., complexation efficacy, complex stability, enzyme recovery and activity of recovered enzyme) of this complex were identified and the pH of the medium, LYZ/SDS molar ratio and the drying parameters were revealed as high-risk factors. A new cost-effective approach based on electrostatic titration was found efficient in the determination of the optimum LYZ/SDS molar ratios at different pH values which resulted in high complexation yield above 95% at pH 4, 6 and 8. The LYZ/SDS molar ratio of 1:8 at pH 8 was found to be the best condition to achieve high complexation yield along with the highest recovery (%) of biologically active enzyme. Moreover, a newly developed and optimized method was proposed to dry such a complex in about three hours using a ventilated oven. The CQAs of the complex dried by this method were comparable to that dried by the commonly used freeze-drying technique which is a time- and energy-demanding technique.

## Figures and Tables

**Table 1:** Two-level full factorial design of the drying conditions in the optimization experiment.

Run	Temperature (°C) (*x*_1_)	Fan Speed * (*x*_2_)
1	25	50
2	40	50
3	25	100
4	40	100

* Set values equal to 50 and 100% fan capacity, respectively.

**Table 2:** Quality target product profiles (QTPPs) of the HIP complexation of LYZ with SDS and the associated critical quality attributes (CQAs).

QTPP	Target	Justification	CQAs	Target	Justification
Lipophilicity	Enhanced lipophilicity	Important to enhance the encapsulation of peptides and proteins in lipophilic micro/nanocarriers. Help in the protection of hydrophilic macromolecules from the destructive effect of organic solvents used during the preparation of micro/nanocarriers [5].	Complexation efficiency	High	To increase the yield of the complexed peptide and consequently the efficiency of the process [11].
			Complex stability	Maximum, During preparation and storage	To achieve high encapsulation efficiency in hydrophobic colloidal carriers and reduce losses [5].
Biological activity	The maximum possible preserved biological activity	Preserving the secondary and tertiary structures of peptides and proteins is crucial for their biological activity [7].	Preserving the secondary structure of the peptide	Maximum possible enzymatic activity	It is crucial to achieve biological activity [7].
Reversibility	Reversible dissociation upon contact with the biological fluids	Dissociation of the complex in the biological fluids is important to release peptide/protein to be biologically available [3].	Recovery (dissociation of the complex)	Maximum dissociation of the peptide	To obtain a free form of the peptide in biological fluids for therapeutic activity [3].

**Table 3:** Weight losses on TGA thermograms and DSC thermogram peaks identified for LYZ, SDS, oven-dried and freeze-dried LYZ/SDS HIP complexes.

Ingredients	Weight Loss (%) (Heating from 25 °C to 500 °C)	DSC Thermogram Peaks
SDS	66.54	Endothermic dehydration peak at 109.67 °C
		Sharp endothermic peak at 199.48 °C (melting point)
		Decomposition peaks at 219.34 (endothermic), 238.86 °C (exothermic) and 275.36 °C (endothermic)
LYZ	65.42	Broad endothermic peak from 38.72 °C to 145.45 °C due to water removal
		Endothermic peak at 201.59 °C due to denaturation (Tm)
		Endothermic decomposition peak at 307.74 °C
Oven-dried HIP	58.93	Broad endothermic peak at 105.40 °C due to water removal
		Endothermic peak at 196.76 °C
		Decomposition peak at 277.29 °C
Freeze-dried HIP	72.23	Broad endothermic peak at 102.60 °C due to water removal
		Endothermic peak at 207.72 °C
		Endothermic decomposition peak at 276.43 °C

## Data Availability

Data are available upon request.

## Supplementary Materials

The following supporting information can be downloaded at: https://www.mdpi.com/article/10.3390/pharmaceutics16050589/s1, Figure S1. The Percentage of the complexed LYZ with SDS at a 1:6 molar ratio at different pH values (average of three replicates). Figure S2. The interrelationship between the quality target product profile (QTTP) and the critical quality attributes (CQA) of the peptide/protein HIP complex. Figure S3. Risk estimation matrix (REM) showing the interdependence rating between CQAs of the complex and CMAs and CPPs of the preparation method ranking them as high-risk, medium-risk and low-risk parameters. Figure S4. Pareto chart showing the ranking of the critical quality attributes (CQAs) of the peptide/protein HIP complex. Figure S5. Complexation efficiency of the complexes prepared at pH 4, 6, 8 and 10 with the molar ratios obtained from the titration experiment (average of six measurements). Figure S6. Enzymatic activity (%) of the dissociated LYZ from the HIP complex prepared at pH 4 (a), pH 6 (b) and pH 8 (c) (average of three replicates). Figure S7. Enzyme activity (%) of LYZ recovered from the HIP complex prepared at pH 6 (a) and pH 8 (b) and collected at different time points (average of five measurements).
