# Supplementary material for: QbD Approach-Based Preparation and Optimization of Hydrophobic Ion-Pairing Complex of Lysozyme with Sodium Dodecyl Sulphate to Enhance Stability in Lipid-Based Carriers
Source: Pharmaceutics. 2024 Apr 26;16(5):589. doi: 10.3390/pharmaceutics16050589 (PMC11125345; doi:10.3390/pharmaceutics16050589)
Supplement: Supplementary file 1 [file pharmaceutics-16-00589-s001.zip › pharmaceutics-2944528-supplementary.pdf]

Supplementary Materials

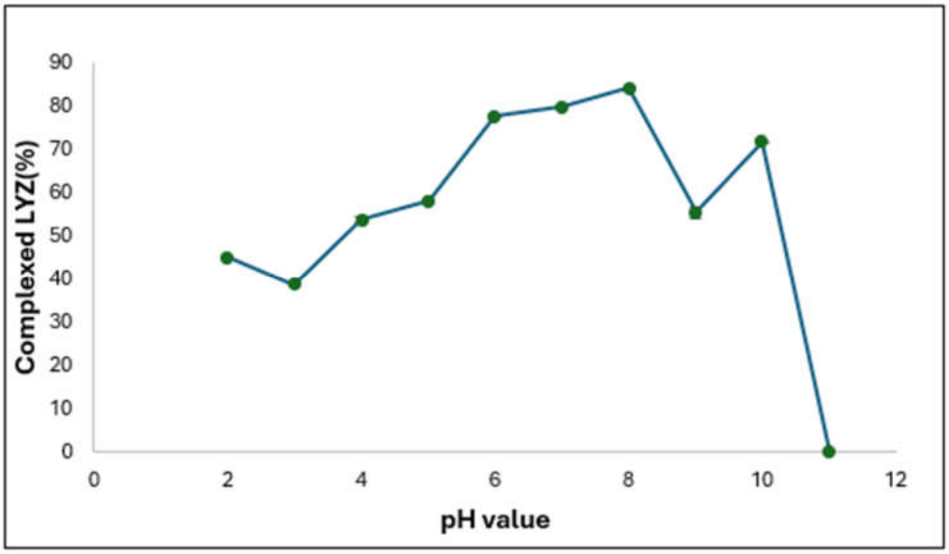

Figure S1. The Percentage of the complexed LYZ with SDS at 1:6 molar ratio at different pH values (average of three replicates).

QTPP-CQA: HIP complex of Lysozyme

CQA \ QTPP	Enhancing the lipophilicity	Preserving the biological activity	Reversibility
High complexation efficiency	High	Medium	Low
Preserving of the secondary structure	Medium	High	Low
Complex stability (during preparation and storage)	High	Low	Low
High recovery (%)	Low	Low	High

Figure S2. The interrelationship between the quality target product profile (QTTP) and the critical quality attributes (CQA) of peptide/protein HIP complex.

Process:	Incubation of protein with IPA								Centrifugation			Drying		
CQAs \ CPP/CMA:	Type of protein/peptide	IPA type	Protein:IPA ratio	Buffering system	pH value	Incubation time	Incubation temperature	Mixing technique	Centrifugation speed	Centrifugation time	Centrifugation temperature	Drying method	Drying temperature	Drying time
High complexation efficiency	Medium	Medium	High	Low	High	Medium	Low	Low	Low	Low	Low	Low	Low	Low
Preserving of the secondary structure	High	Medium	Medium	Low	High	Low	Medium	Low	Low	Low	Low	High	High	High
Complex stability (during preparation and storage)	Medium	Medium	Low	Low	Medium	Low	Low	Low	Low	Low	Low	Medium	Medium	Medium
High recovery (%)	Medium	Medium	Medium	Low	High	Low	Low	Low	Low	Low	Low	Low	Low	Low

Figure S3. Risk estimation matrix (REM) showing the interdependence rating between CQAs of the complex and CMAs and CPPs of the preparation method ranking them as high-risk, Medium-risk and Low-risk parameters.

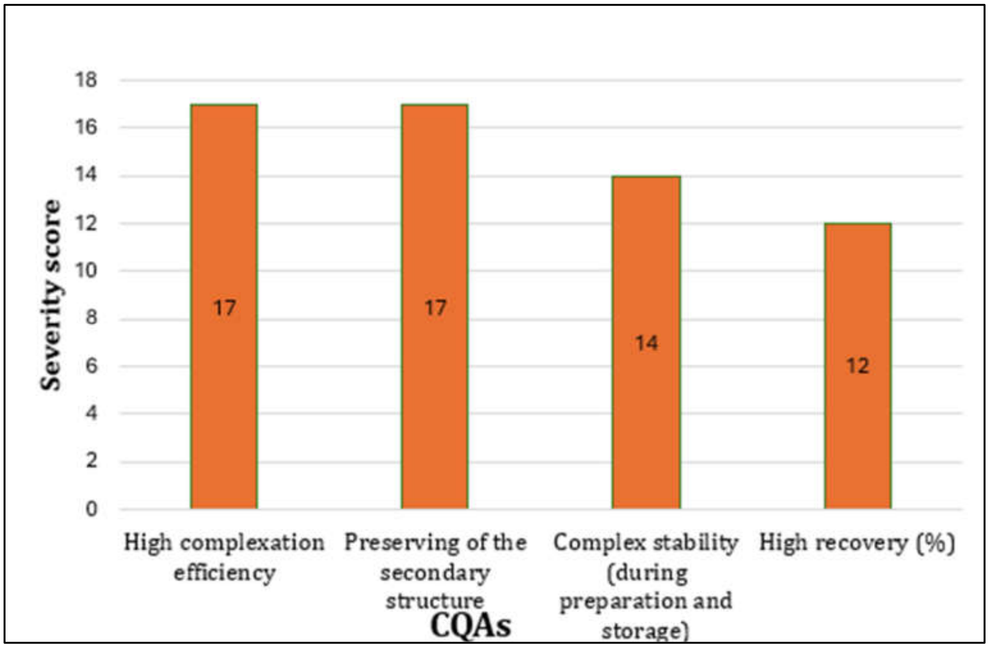

Figure S4. Pareto chart showing the ranking of the critical quality attributes (CQAs) of peptide/protein HIP complex.

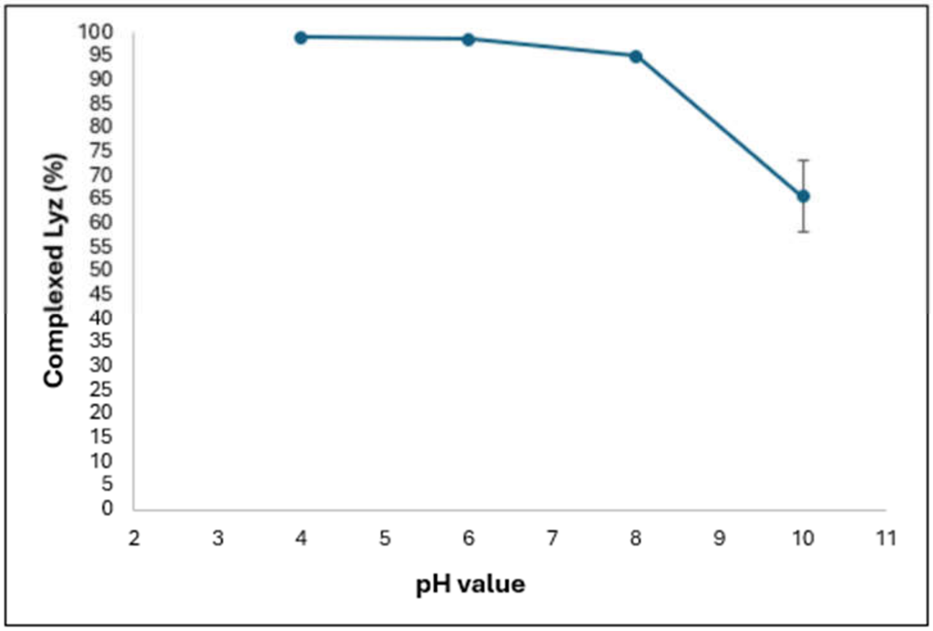

Figure S5. Complexation efficiency of the complexes prepared at pH 4, 6, 8 and 10 with the molar ratios obtained from the titration experiment (average of six measurements).

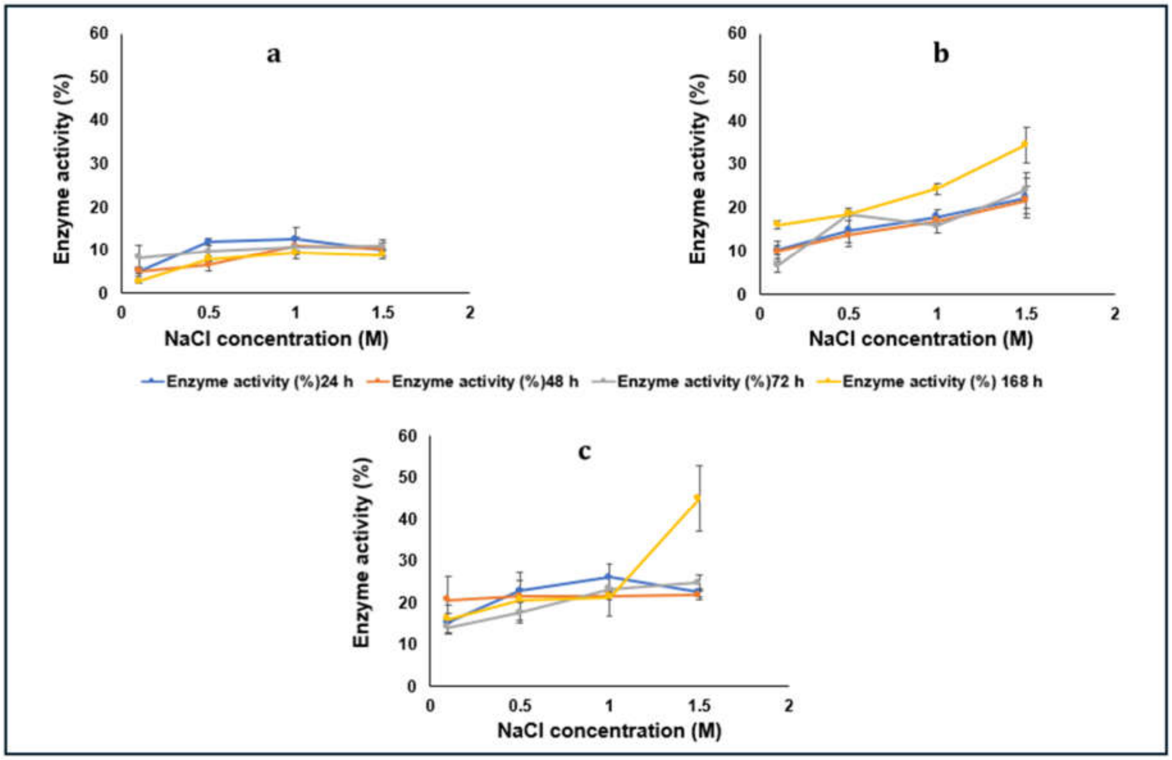

Figure S6. Enzymatic activity (%) of the dissociated LYZ from the HIP complex prepared at pH 4 (**a**), pH 6 (**b**) and pH 8 (**c**) (average of three replicates).

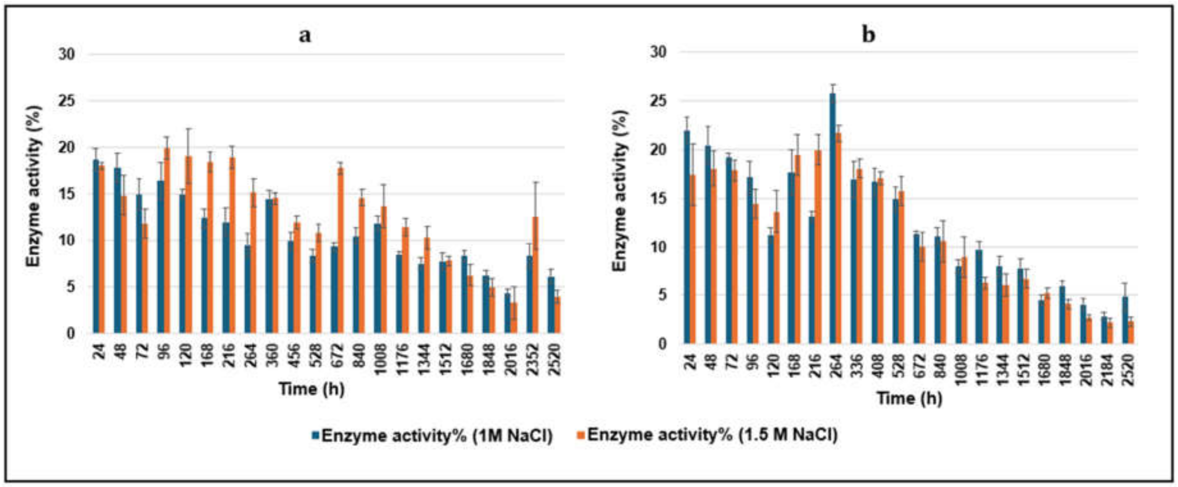

Figure S7. Enzyme activity (%) of LYZ recovered from the HIP complex prepared at pH 6 (**a**) and pH 8 (**b**) and collected at different time points (average of five measurements).
